# Uncovering Systemic Dynamics through an Integrated WEFE Nexus Index across 21st Century Futures

**DOI:** 10.1021/acs.est.5c11740

**Published:** 2026-02-05

**Authors:** Zeynep Özcan, Emre Alp

**Affiliations:** Department of Environmental Engineering, Middle East Technical University, Üniversiteler Mahallesi, Dumlupınar Bulvarı No:1, Çankaya, Ankara 06800, Turkey

**Keywords:** water–energy–food–ecosystem nexus, climate change, environmental flows, WEAP–LEAP model, water policy, shared socioeconomic pathways

## Abstract

Achieving sustainability under accelerating climate and socioeconomic pressures requires moving beyond siloed sectoral management toward a system-thinking approach. The water–energy–food–ecosystem (WEFE) Nexus offers a holistic lens, yet most applications remain conceptual, short-term, or treat ecosystems as external constraints. This study operationalizes the WEFE Nexus by embedding ecosystems as a coequal, quantified pillar through a hydrologic-regime-based method, since streamflow is a master variable shaping riverine ecosystem health. Long-term foresight is incorporated via dynamically downscaled climate projections and Shared Socioeconomic Pathways within a coupled water and energy systems (WEAP–LEAP) model. Applied to the semiarid Sakarya Basin in Türkiye, the framework evaluates three future periods (2020–2030, 2055–2065, and 2090–2100) across seven subbasins. Results show systemic trade-offs: municipal water security remains high (>90%), but ecosystem integrity and renewable energy goals are consistently compromised. Overall, WEFE Nexus Index values (0.53–0.86) show significant spatial disparities, with arid upstream regions consistently underperforming. Strikingly, SSP2 (business-as-usual) and SSP5 (fossil-fueled growth) yield nearly identical outcomes, underscoring the systemic unsustainability of current trajectories. This framework advances nexus assessment from theory to practice by integrating reproducible metrics, scenario planning, and spatial modeling, creating a practical tool for developing adaptive and resilient sustainability strategies.

## Graphical abstract

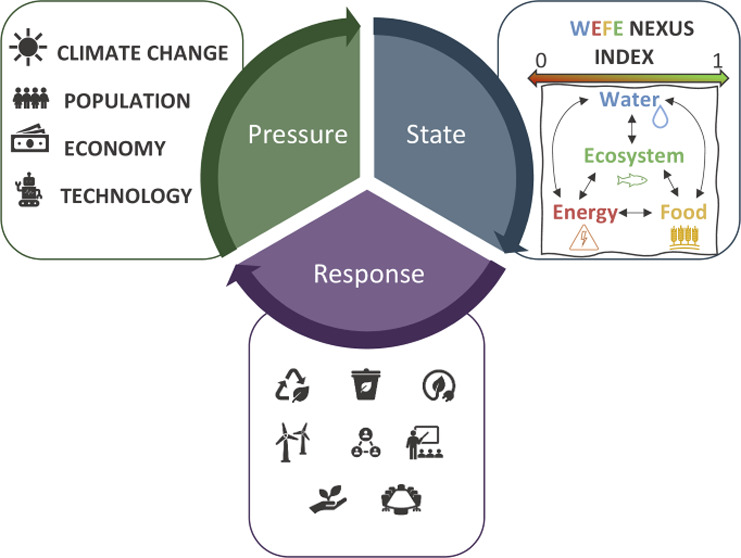

## 1 Introduction

Climate change, population growth, and rising resource demands are intensifying global concerns around the long-term availability and security of water, energy, and food. By 2050, global freshwater demand is expected to increase by 55%, energy use by 50%, and agricultural yields may decline by up to 25%, raising alarms about sustainable development.^1–3^ These pressures extend beyond sectoral limits, threatening the ecosystem integrity, public health, and political stability. Yet despite decades of recognition, progress remains limited: for example, SDG 2 (food security), SDG 6 (clean water and sanitation), SDG 7 (affordable and clean energy), SDG 13 (climate action), and SDG 15 (life on land) all exhibit persistent gaps, with none of the SDG 6 water targets on track as of 2022 and only about 40% of EU surface waters achieving “good” ecological status under the Water Framework Directive.^4,5^ The shortfall reflects the persistence of siloed, sector-by-sector management. Major policies, such as the UN 2030 Agenda, the US Clean Water Act, the EU Water Framework Directive, and Türkiye’s Environmental Law, generally address water, energy, food, and ecosystems as separate entities. This fragmentation undermines their collective effectiveness and highlights the need for systems thinking approaches that explicitly address cross-sector interactions, trade-offs, and feedbacks.^4,6^

The water–energy–food (WEF) Nexus framework has emerged in the past decade as a valuable tool for capturing these interconnections.^7–9^ Nexus research has been applied to evaluate synergies, conflicts, and interdependencies across sectors using scenario modeling,^10–12^ policy analysis,^13–16^ and indicator-based assessments.^17–20^ These contributions highlight the value of integrated frameworks for anticipating cross-sector dynamics and supporting sustainability, though most applications do not fully capture the complexity of coupled human–natural systems.^21–24^

Several limitations stand out. First, many studies remain confined to pairwise or sector-specific analyses, such as water–energy trade-offs or energy–food linkages, rather than fully integrated assessments of cumulative risks.^21,25^ Second, the temporal scope is often limited. Assessments frequently rely on present-day or short-term historical conditions, with minimal integration of long-term socioeconomic and climate futures.^26^ This restricts their relevance for policy and planning under uncertainty, particularly in regions where external stressors are expected to intensify. Third, while indicator-based approaches are proliferating, they often suffer from nonstandardized normalization, weighting, and aggregation methods, hindering comparability across cases.^17,20,21^ Furthermore, although some scenario-based studies now integrate Shared Socioeconomic Pathways (SSPs) and climate projections, few offer a systematic, multiscenario evaluations that span both time and space.^25,27^

A particularly significant gap lies in the treatment of ecosystems. In most Nexus studies, ecosystems are considered external constraints or passive beneficiaries of resource decisions rather than coequal system components. This bias risks prioritizing resource security in ways that degrade ecological integrity, thereby eroding long-term resilience.^28–30^ Empirical reviews confirm this weakness: ecosystems are often represented only through proxy variables in optimization studies,^31^ reduced to descriptive accounts without reproducible methods,^21^ or marginalized relative to water, energy, and food sectors.^32^ The water–energy–food–ecosystem (WEFE) Nexus concept has been proposed to correct this imbalance by embedding ecosystems as a fourth pillar.^33–37^ However, operationalizing this expansion remains rare. Most WEFE studies still rely on conceptual framings with limited analytic rigor or spatially explicit, scenario-responsive applications.^27^ For example, Lucca et al. (2023)^27^ reviewed 142 WEFE-related studies in the Mediterranean and found that WEFE research is still dominated by partial interlinkages (particularly water–energy), while the expansion to additional components and explicit feedback representation remains limited. Likewise, Endo et al. (2020)^38^ showed that fully integrated WEFE implementations are still scarce compared with WEFE-focused studies, with relatively few mature methods available for operational WEFE assessment. More recently, Mooren et al. (2025)^35^ emphasized that WEFE has largely evolved as a governance and conceptual framework and proposed structured approaches to improve operationalization, underscoring the need for more consistent and decision-relevant implementation.

This study addresses these gaps by advancing the WEFE Nexus from conceptual framing to operational application. We introduce a hydrologic-regime-based method to represent ecological integrity alongside water, energy, and food indicators; integrate long-term foresight through dynamically downscaled climate projections and SSPs; and apply a dynamically coupled WEAP–LEAP model across multiple subbasins of the Sakarya Basin, Türkiye. Accordingly, this study has four specific objectives: (i) to develop an operational WEFE Nexus framework that treats ecosystems as a coequal pillar using hydrologic-regime-based ecological indicators; (ii) to incorporate long-term climate and socioeconomic futures across 21st century time scales (near-, mid-, and far-century) into WEFE assessment through downscaled climate projections and SSPs; (iii) to implement a spatially explicit, multiscenario WEAP–LEAP modeling framework for the Sakarya Basin subbasins; and (iv) to construct a transparent and transferable WEFE Nexus Index for benchmarking cross-sector sustainability across time and space and informing adaptive, ecosystem-informed resource planning. The resulting WEFE Nexus Index is a transparent and transferable tool for benchmarking systemic sustainability over time and space. By making ecosystems a quantified pillar and combining scenario foresight with cross-sector modeling, this study advances the nexus approach and delivers actionable insights for adaptive, ecosystem-based strategies amid 21st century uncertainty.

## 2 Materials and Methods

### 2.1 Study Area

The Sakarya Basin, located in northwest Türkiye (Figure 1), covers approximately 7% of the national land area, with a drainage area of about 63,000 km^2^. It supports a population of roughly 7.5 million, around 9% of Türkiye’s total,^39^ and plays a pivotal role in the country’s agricultural production, energy generation, and industrial development. As such, it exemplifies the complex interdependencies and security challenges of the WEFE Nexus under climate and socio-economic pressures.

**Figure 1:**
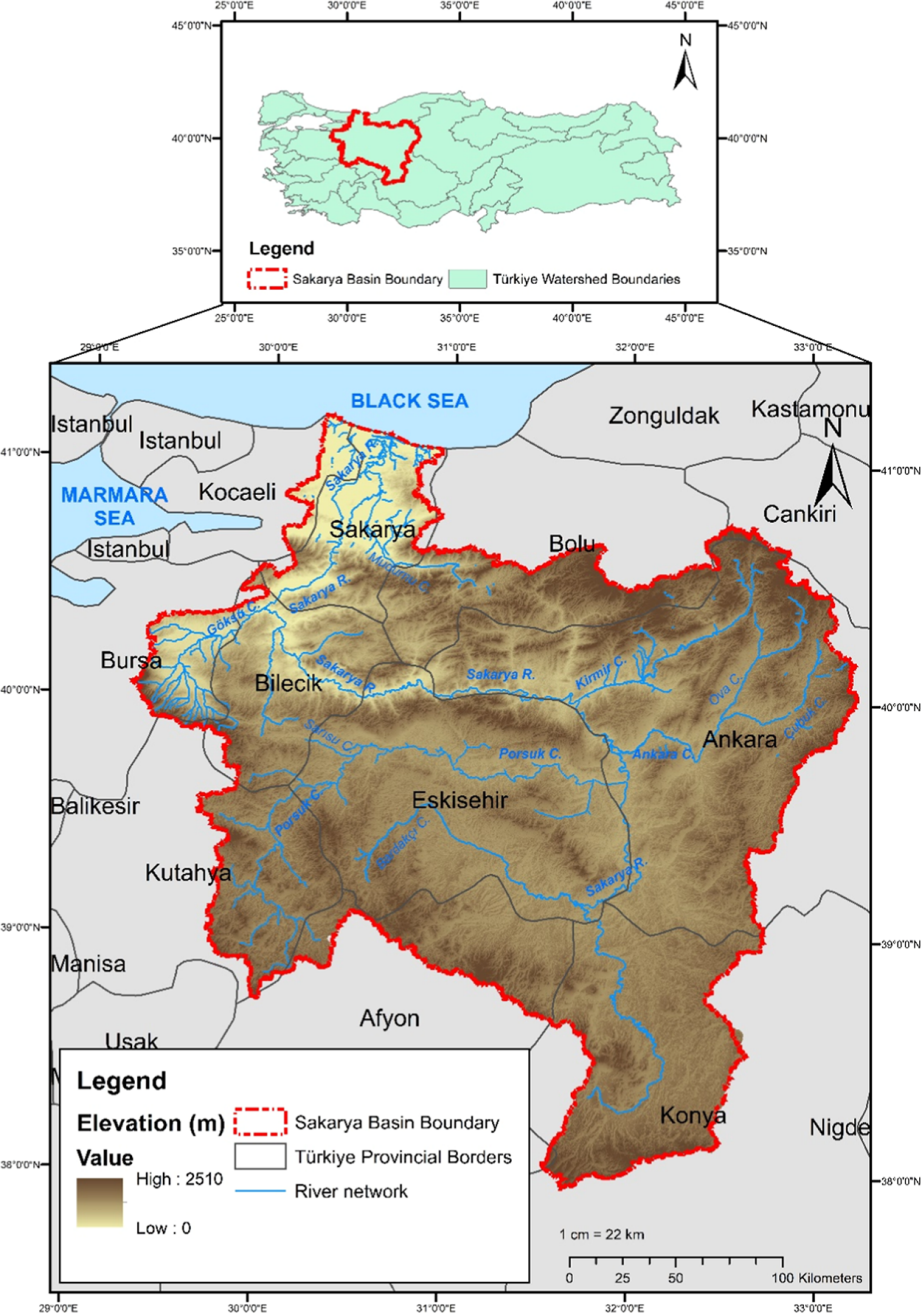
Location of Sakarya Basin in Türkiye, borders of provinces, and digital elevation map.

Despite ongoing industrialization, the basin remains predominantly agricultural, with agricultural lands covering 53% of its total area.^40^ This heavy reliance on irrigation-based farming makes it highly sensitive to hydrologic variability and water scarcity, which are expected to intensify under climate change. Meanwhile, the region also hosts 20 organized industrial zones and nearly 100 power plants, generating over 35,000 GWh of electricity annually, primarily from fossil fuels. This high demand for water and energy, coupled with limited precipitation (annual average ∼479 mm), puts the basin at risk of resource conflict, ecological degradation, and policy trade-offs.

Administratively and hydrologically, the Sakarya Basin is divided into seven subbasins: Upper Sakarya, Porsuk, Ankara, Kirmir, Middle Sakarya, Göksu, and Lower Sakarya. These subbasins differ in land use, population, economic activity, and hydrologic regimes, offering a diverse set of conditions for evaluating spatially differentiated WEFE Nexus performance. For instance, while the Upper Sakarya subbasin suffers from aridity and agricultural stress, the Lower Sakarya receives cumulative upstream pressures, caused by intensive upstream activities, such as irrigation withdrawals, hydropower operations, and industrial discharges in subbasins like Ankara and Porsuk.

These characteristics make the Sakarya Basin an ideal case study for developing and applying a comprehensive WEFE Nexus evaluation framework. It provides a realistic testing ground for assessing long-term sustainability under climate change, fossil-fuel-reliant energy production, increasing food demand, and vulnerable ecosystem conditions. Table 1 provides an overview of the subbasins, detailing their land use, hydrology, population, industrial GDP, and projected 21st century climate trends.

**Table 1:** Summary of Key Characteristics of the Subbasins Included in This Study^41^

	drainage area (km^2^)	agricultural areas (%)	population	industrial gross domestic product^a^ (million TL)	current state river flow (hm^3^/y)	annual average hydroelectric generation (GWh)	annual average thermal electricity generation (GWh)	21st century precipitation trend^b^	21st century temperature trend^b^
Upper Sakarya	21,354	69.4	416,622	26.273 (Konya)	722	-	198	decreasing	increasing
Porsuk	10,825	50.4	1,002,397	26.273 (Eskişehir)	256	-	4008	decreasing	increasing
Ankara/Kirmir	7165/4600	64.2/32.6	4,572,464/94,775	83.019 (Ankara)	432/371	-	6141	decreasing	increasing
middle Sakarya	12,093	31.6	343,357	6.586 (Bilecik)	838	1388	6633	decreasing	increasing
Göksu	2435	49.8	288,985	81.188 (Bursa)	564	126	179	decreasing	increasing
lower Sakarya	4830	50.3	805,421	23.643 (Sakarya)	2048	173	16,059	decreasing	increasing

a For the gross domestic product indicator, the value of the largest province in terms of population within the borders of the subbasin was used.

b Bold text means the trend is statistically significant.

### 2.2 Proposed WEFE Nexus Framework

To ensure the long-term sustainability of water, energy, food, and ecosystems, it is crucial to identify the root causes of environmental challenges and develop effective strategies to address them. The proposed WEFE Nexus evaluation methodology is embedded in the pressure-state response framework (Figure 2), a widely applied approach for analyzing complex systems. In this framework, the system is evaluated based on three key components: the pressures exerted on the system, the current state of the system, and the responses designed to address these two factors.^42^

**Figure 2:**
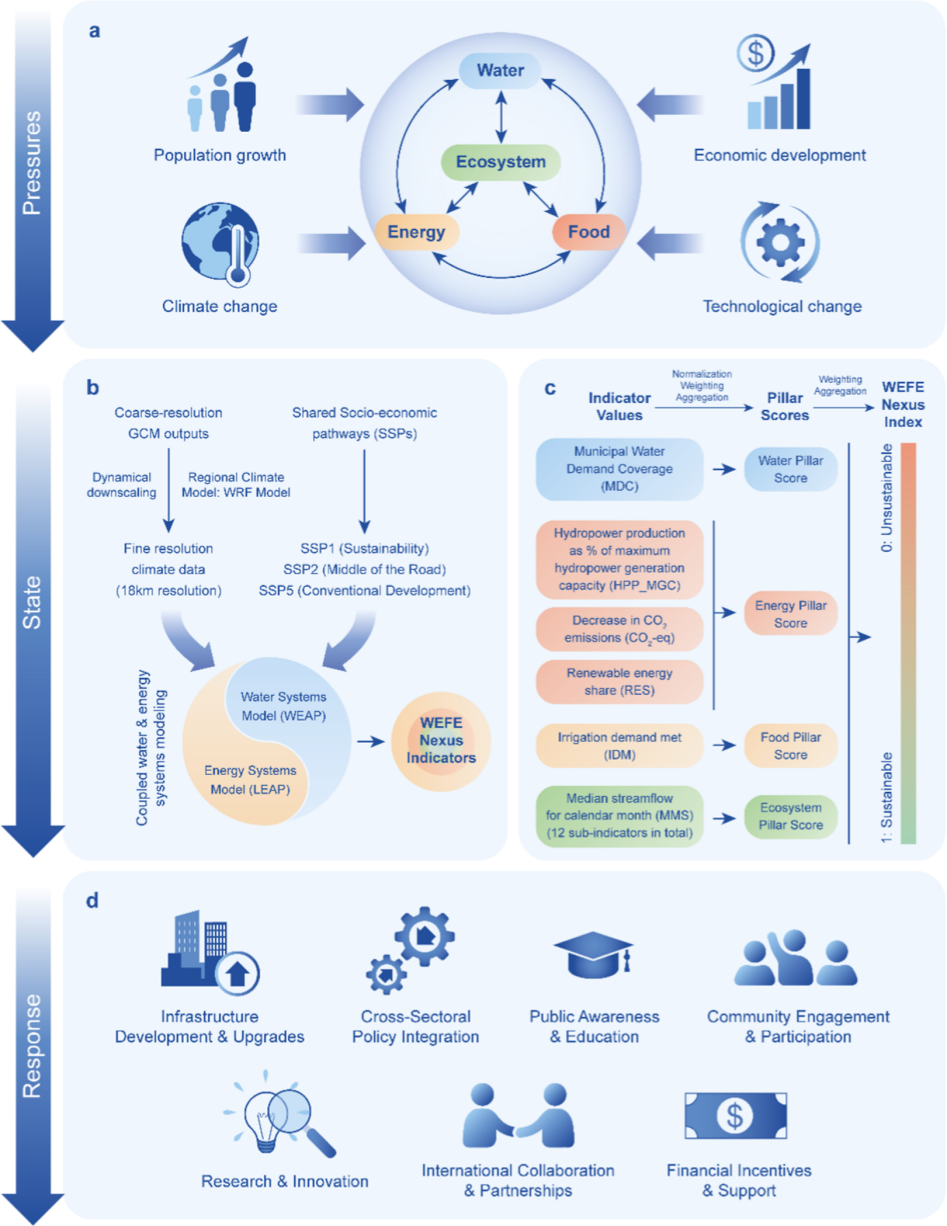
Methodological framework for assessing the WEFE Nexus under climate change and socioeconomic pressures. GCM: general circulation model; WRF: weather research and forecasting model; WEAP: water evaluation and planning system; and LEAP: long-range energy alternatives planning system.

Figure 2 outlines the methodology used in this study. It begins with identifying key pressures on the WEFE Nexus, such as climate change, population growth, economic development, and technological change, to assess their potential impacts and guide intervention priorities (2a). The next step evaluates the state of the WEFE Nexus (Figure 2b,c) by applying a coupled model that integrates water, energy, food, and ecosystems to generate relevant indicators. Finally, responses are explored (Figure 2d), focusing on policies and strategies that support sustainability and resilience. Further methodological details are provided in the sections below.

#### 2.2.1 Pressures (Figure 2a)

The WEFE Nexus framework addresses a range of external pressures that shape system sustainability and resilience. Four broad, overlapping categories, i.e., climate change, population growth, economic development, and technological change, are used to structure these drivers. While not exhaustive, they capture key dynamics represented in SSP-based modeling, which are frameworks that explore different global development scenarios based on future socioeconomic trends.^43,44^ These pressures include:

i Climate change alters precipitation and temperature patterns, affecting water availability, energy demand, agricultural productivity, and ecosystem services.^27,36^

ii Population growth increases demand for water, energy, and food resources.^23^
iii Economic development intensifies pressure on water and energy systems.^45^
iv Technological change can improve resource efficiency but may also introduce new environmental risks.^46,47^

The operationalization of these pressures is grounded in the use of SSPs and dynamically downscaled climate projections, which inform a broad set of input parameters in the coupled WEAP–LEAP model, an integrated modeling platform used to assess cross-sector interactions across water, energy, food, and ecosystems. Key aspects, such as land use change, sectoral water and energy demands, and water reuse, are adjusted in alignment with SSP narratives. For example, resource endowment and infrastructure expansion are reflected through scenario-specific assumptions about supply systems, technological adoption, and demand trajectories. Similarly, although governance or institutional capacity is not explicitly modeled, its implications are indirectly embedded within the socioeconomic assumptions of each SSP.

#### 2.2.2 State: Integrated Modeling (Figure 2b)

This study integrates future climate and socio-economic conditions into a coupled WEAP–LEAP modeling framework. Climate projections were dynamically downscaled using the WRF model (Section 2.4), while socioeconomic influences were represented through SSPs,^43,48^ specifically SSP1 (sustainability), SSP2 (middle of the road), and SSP5 (conventional development). SSPs describe potential global trajectories in the absence of new climate policies and are designed to complement representative concentration pathways (RCPs), which represent greenhouse gas concentration scenarios.^49^ Accordingly, RCP 4.5 (low emissions) and RCP 8.5 (high emissions) were paired with the SSPs to evaluate a range of plausible futures.

Using both qualitative narratives and quantitative projections, key socioeconomic parameters were adjusted within the WEAP–LEAP model (Section 2.6). The integration of WEAP and long-range energy alternatives planning (LEAP) (Section 2.3) enables dynamic simulation of cross-sector interactions across water, energy, food, and ecosystem components, capturing feedback and trade-offs critical to WEFE Nexus analysis.

#### 2.2.3 State: WEFE Nexus Index Development (Figure 2c)

The coupled WEAP–LEAP model was used to generate key indicators that assess the WEFE Nexus performance across multiple future scenarios. By integration of climate projections and socioeconomic parameters, the model provides a comprehensive view of system dynamics under varying conditions. These indicators convert complex interactions into measurable outcomes, enabling a systematic, scenario-based evaluation.

The food pillar is explicitly represented through agriculture, which dominates land use and water consumption in the Sakarya Basin. Accordingly, irrigation demand met (IDM) (%) is used as the food pillar’s indicator, capturing both water availability and the system’s ability to support agricultural productivity under changing conditions. The use of irrigation demand deficit or fulfillment as a proxy for agricultural production is common in the literature.^50^ The municipal water demand coverage (%) reflects the system’s capacity to supply drinking water, which is especially critical in densely populated areas such as Ankara. In the energy pillar, hydropower production (%) indicates the degree to which hydropower potential is utilized, while CO_2_ emission reduction (%) reflects progress toward national climate goals, particularly relevant given that electricity generation is Türkiye’s largest source of CO_2_ emissions.^51^ The renewable energy share (RES) (%), which represents the proportion of renewable energy in total electricity production, addresses energy security and supports national targets for reducing fossil fuel dependency. Finally, the ecosystem pillar is assessed using median monthly streamflow, which serves as a proxy for environmental flow conditions and hydrologic health across the year (Section 2.5).

The following presents the set of indicators employed in the analysis, each accompanied by its corresponding calculation formula:

##### 2.2.3.1 Municipal Water Demand Coverage (MDC) (%)

To calculate MDC, the WEAP–LEAP model results and eq 1 are used. First, the supply requirement (m^3^) of and supply delivered (m^3^) to each municipal demand site node in the relevant subbasin are obtained. Then, the total amount of supply requirement and supply delivered is calculated by summing up all demand site nodes. Finally, the whole watershed municipal water demand coverage is calculated as it is given in eq 1.

1 $$Municipal\;water\;demand\;coverage\left(\%\right)={\frac{\sum_{i}\sum_{t}supply\;delivered\left(m^{3}\right)}{\sum_{i}\sum_{t}supply\;requirement\left(m^{3}\right)}}^{*}100$$

where *i* represents the number of municipal demand site nodes and *t* is the number of months for the related period, i.e., 108 months for the near century (2022–2030), midcentury (2057–2065), and far century (2092–2100).

##### 2.2.3.2 Hydropower Production as % of Maximum Hydropower Generation Capacity (HPP_MGC)

To calculate HPP_MGC, first, the maximum electricity generation that can be produced in a year from the hydropower plants is calculated by using their installed capacities. Then, the average annual value produced from the hydroelectric power plants during the simulation period is obtained. Finally, HPP_MGC is calculated as follows (eq 2)­

2 $$Hydro.prod.\text{as}\;\%\;of\;\max.gen.capacity\;of\;HPPs=\frac{ave.total\;hydro\left(GWh/year\right)}{hydro.\max.generation\;capacity\left(GWh/year\right)}\times 100$$

##### 2.2.3.3 CO_2_ Emission Reduction (CO_2__EG) (%)

To calculate CO_2__EG, first, the annual average CO_2_ emissions in the baseline period (2004–2017) were calculated for each subbasin. Next, the annual average CO_2_ emissions in each future period segment, i.e., near, mid, and far centuries, were calculated in each subbasin. Finally, the CO_2__EG is calculated as follows (eq 3)­

3 $$Decrease\;in\;{CO}_{2}\;emissions\left({CO}_{2}\text{\_}EG\right)=\frac{ave.{CO}_{2}\left(baseline\right)-ave.{CO}_{2}\left(future\;period\right)}{ave.{CO}_{2}\left(baseline\right)}\times 100$$

##### 2.2.3.4 Renewable Energy Share (%)

To calculate RES, first, the average annual electricity generation from the renewable energy plants throughout the relevant period is calculated based on the LEAP model results. Then, the average annual total electricity generation for the same period is calculated. Finally, the RES is calculated as follows (eq 4)­

4 $$Renewable\;energy\;share\left(RES\right)\left(\%\right)=\frac{ave.annual\;electricity\;generation\left(renewable\;plants\right)}{ave.annual\;electricity\;generation\;\left(\text{all}\;power\;plants\right)}\times 100$$

##### 2.2.3.5 Irrigation Demand Met (%)

To obtain the IDM (%), first, the sum of the total amount of irrigation shortfall (m^3^) in all catchment branches in the watershed is calculated, and it is divided by the sum of the total theoretical catchment irrigation demand (m^3^) in all catchment branches in the study watershed. This calculation is performed on catchments with irrigated agriculture. In this calculation, an irrigation demand deficit is found. To calculate IDM, the calculation given in eq 5 is employed

5 $$Irrigation\;demand\;met\left(\%\right)=\left(1-\frac{\sum_{i}\sum_{t}irrigation\;short\;fall\left(m^{3}\right)}{\sum_{i}\sum_{t}theo.\;catchment\;irrigation\;demand\left(m^{3}\right)}\right)\times 100$$

where *i* represents the number of catchments with irrigated agriculture and *t* is the number of years for the related period, i.e., 9 years for the near century (2022–2030), mid century (2057–2065), and far century (2092–2100).

##### 2.2.3.6 Median Discharges for Each Calendar Month (MMS) (m^3^/sec) (12 Indicators in Total)

The assessment of ecosystem pillar indicators and the ecosystem pillar is explained in detail in 2.5.

Once all WEFE indicators are calculated, they are incorporated into the WEFE Nexus Index, a composite unitless metric designed to quantify overall system sustainability. The index integrates the water, energy, food, and ecosystem pillars by first normalizing each indicator to a common 0–1 scale, ensuring comparability regardless of units or magnitude. The normalized indicators within each pillar are then aggregated to produce pillar-level scores, and these four scores are combined using equal weighting, reflecting the coequal importance of each sector in the WEFE framework. The final index ranges from 0 (unsustainable conditions) to 1 (highly sustainable conditions), providing a standardized basis for comparing WEFE performance across subbasins, scenarios, and future time periods. Detailed procedures for normalization, weighting, and aggregation are provided in 2.7.

#### 2.2.4 Response (Figure 2d)

The responses to the proposed WEFE Nexus evaluation methodology are guided by the sustainability status indicated by the WEFE Nexus Index. While the index offers a quantitative basis for assessment, specific thresholds distinguishing “high” or “low” levels of sustainability and resilience are not universally defined. As the WEFE Nexus Index is designed to be context-sensitive, threshold definitions for interpreting sustainability levels (e.g., high or low resilience) are not predefined but should be established in each application in consultation with local stakeholders. This ensures that the response actions derived from the index are relevant to the specific needs, priorities, and decision-making processes of the study area. In practice, higher index values may indicate a relatively balanced nexus performance, where the focus can remain on maintaining and fine-tuning existing policies. Conversely, lower values may signal critical imbalances that call for more substantial, targeted interventions. These may include infrastructure upgrades, cross-sectoral policy integration, public awareness and education, community engagement, research, and innovation, financial support mechanisms, and international collaboration.

### 2.3 WEAP–LEAP Model Setup

Addressing the complex interrelations among the nexus components and the effects of external factors on the nexus requires tools capable of representing both natural and social systems. To address questions regarding WEFE nexus security and to analyze the interlinkages among its components, an integrated WEAP–LEAP model is employed. This integrated model has been widely applied in various studies for diverse purposes.^52,53^

The water evaluation and planning system (WEAP)^54^ is a conceptual hydrological model designed to operate on the basic principle of a water balance. It can be applied to municipal and agricultural systems, individual watersheds, and complex transboundary river basin systems. Developed by the Stockholm Environment Institute (SEI), WEAP serves as a tool for integrated water resource planning and management. It can solve multisectoral water allocation problems based on demand priorities and supply preferences. WEAP can represent various aspects of water systems, including water resources such as surface water and groundwater; water demands such as urban, hydropower, irrigation, and environmental flows; and water infrastructures such as reservoirs, canals, and wells.

The LEAP system, as the name suggests, is a long-term integrated energy planning and greenhouse gas (GHG) mitigation modeling system. LEAP can be used to track energy consumption, production, and resource extraction across all economic sectors. It is capable of accounting for GHG emissions from both energy and nonenergy sources, as well as analyzing emissions of local and regional air pollutants and short-lived climate pollutants.^55^

Figure 3 illustrates the conceptual representation of the WEAP–LEAP model integration including the broadly categorized inputs of both models. The WEAP and LEAP models are dynamically coupled using WEAP value and LEAP value functions, which enable two-way data exchange between water and energy systems. For instance, water requirements for energy production, such as cooling water requirements for thermal power plants and hydropower generation, are modeled in WEAP and integrated into the energy model. Hydropower is modeled in WEAP and then integrated into the LEAP model. LEAP addresses total electricity demand by dispatching its power mix. This includes all available electricity resources for a given time step, including WEAP’s estimate of available hydropower alongside other electricity sources such as coal, natural gas, and nuclear power.^53^ By linking these two models, the integrated tool enables the analysis of interlinkages among nexus components.

**Figure 3:**
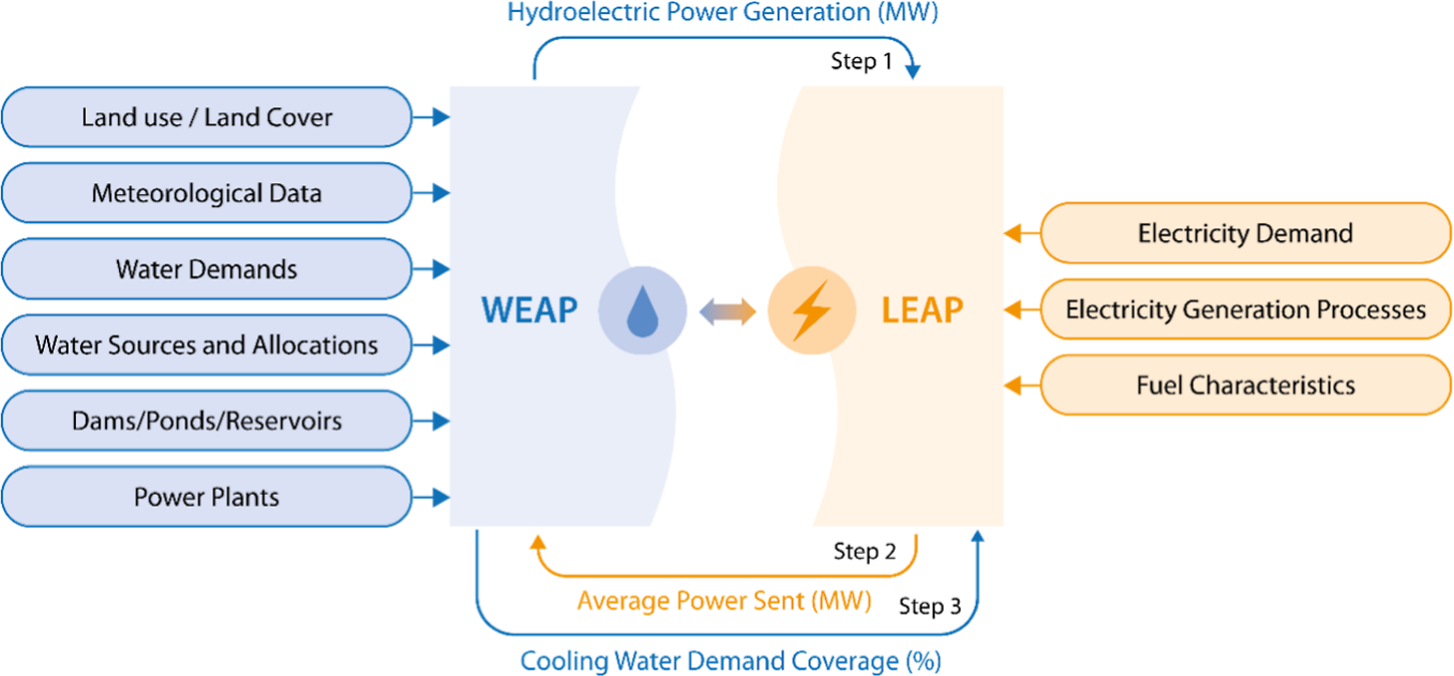
Conceptual representation of WEAP–LEAP integration. WEAP: water evaluation and planning system; LEAP: long-range energy alternatives planning system.

Model calibration and validation were conducted using (i) streamflow values, (ii) reservoir volumes, and (iii) electricity generation. Gauging stations at the outlet of each subbasin were used for calibration when data were available; otherwise, alternative gauging stations were used. The performance of the WEAP–LEAP model was assessed using *R* ^2^, Nash Sutcliffe Efficiency (NSE), and Percent Bias (PBIAS) following the general performance ratings for a monthly time step.^56^ The list of calibration parameters is provided in Table S1. We report that the model demonstrated satisfactory performance^41^ (Table S2).

### 2.4 Dynamical Downscaling of Climate Projections

Future climate data for this study were generated through dynamical downscaling by using the WRF regional climate model. First, the model was calibrated by downscaling ERA-Interim reanalysis data (80 km resolution) and comparing basin-averaged outputs with observations. Calibration was performed for 2010, and the model was then used to reconstruct historical climate conditions from 2009 to 2018.

Once satisfactory performance was achieved, the validated WRF model was used to downscale four future climate projections for the Sakarya Basin to an 18 km resolution suitable for hydrologic modeling. These projections were derived from two GCMs (CCSM4 and MIROC5) under two emission scenarios (RCP of 4.5 and RCP of 8.5). After bias correction was applied, daily outputs, i.e., precipitation, temperature, wind speed, and relative humidity, were aggregated to monthly values and used as climate inputs in the coupled WEAP–LEAP model for scenario-based simulations. The selected projections capture a range of plausible climate futures, with most subbasins exhibiting statistically significant declines in precipitation alongside a consistent increase in temperature. Notably, the MIROC5_RCP8.5 projection indicates the highest warming, particularly in the late century. These changes are spatially heterogeneous across the basin, driven by subbasin-specific topography and location, highlighting the necessity of localized climate assessments.^57^

### 2.5 Assessment of Environmental Flows in Sakarya Basin: Ecosystem Nexus Component

River ecosystems are fundamentally shaped by the quantity, timing, and variability of streamflow. Maintaining natural hydrologic regimes is therefore critical to sustaining ecological integrity, supporting habitat availability, biogeochemical processes, and aquatic biodiversity.^58,59^ In this study, streamflow-based indicators are used to explicitly represent the ecosystem pillar within the WEFE Nexus framework, enabling the quantification of hydrological alteration under alternative future scenarios.

Streamflow is widely recognized as a master variable governing riverine ecosystem integrity because it integrates climatic signals, watershed processes, and anthropogenic regulation into a single controlling driver of aquatic conditions.^58^ The natural flow regime, characterized by its magnitude, frequency, duration, timing, and rate of change, directly shapes water quality, physical habitats, energy fluxes, and biotic interactions, which together determine the ecological integrity of river systems.^59^ Regular and seasonal flow variability supports life-history strategies, sediment transport, lateral and longitudinal connectivity, and habitat renewal, while altered regimes often disrupt these processes, favor invasive species, reduce aquatic biodiversity, and degrade ecosystem functioning.^60^ Although ecosystem integrity is a multidimensional concept involving additional components, such as geomorphology, riparian vegetation, and sediment dynamics, the present study explicitly focuses on the aquatic ecosystem, where hydrological processes serve as the primary control and are most directly linked to the modeling framework employed.

The assessment is based on a modified application of the indicators of hydrologic alteration (IHA) method, developed by Richter et al. (1997, 1996) as part of the range of variability approach (RVA).^61,62^ The RVA framework defines river management targets based on the natural flow regime and evaluates the extent to which postimpact flows deviate from these reference conditions. It originally included 33 hydrologic indicators grouped by ecological relevance; however, due to the use of monthly time steps in this study, only Group 1 parameters (monthly flow magnitudes) were employed. These indicators are directly linked to ecosystem functions such as aquatic habitat availability, soil moisture, and thermal regulation.^63^

Hydrological alteration was evaluated across seven river segments, with each corresponding to a subbasin outlet in the Sakarya Basin. A five-step procedure was used:

1 Pre- and postimpact flow conditions were defined for each scenario and period. Preimpact conditions were simulated by removing all anthropogenic influences, while postimpact conditions included all relevant demands and infrastructure.
2 Monthly mean streamflows were calculated for each year under both conditions.
3 Interannual statistics were computed for each calendar month using median and interquartile range (IQR) values over the evaluation periods (baseline: 2003–2017; near century: 2022–2030; midcentury: 2057–2065; end century: 2092–2100).
4 Median monthly streamflows under postimpact conditions were compared with the IQR of the corresponding natural regime to quantify deviation.
5 A composite hydrologic alteration index was derived by averaging indicator scores across all 12 months.

The scoring follows a nonparametric approach to account for the non-normality of hydrologic data and reduce sensitivity to outliers.^64^ An indicator score of 1 is assigned when the postimpact monthly median lies within the IQR of natural conditions; scores decline linearly to 0 if the value approaches or exceeds the lower or upper extremes (*Q*_min_ or *Q*_max_) of the natural range. It is important to note that this linear scaling reflects a normalization choice for comparative sustainability assessment and does not imply a linear ecological response to hydrological alteration. This scoring procedure is provided in Table 2, which shows how median monthly streamflows are evaluated against the natural reference range to quantify the degree of hydrological alteration. The resulting composite index, ranging from 0 (highly altered) to 1 (unaltered), serves as the normalized input for the ecosystem pillar within the WEFE Nexus Index.

**Table 2:** Hydrological Alteration Scoring and Sustainability Classification Based on Deviations from the Natural Flow Regime^a^

MMS ≤ *Q*_min_	*Q*_min_ ≤ MMS ≤ Q_25_	Q_25_ ≤ MMS ≤ Q_75_	Q_75_ ≤ MMS ≤ *Q*_max_	MMS ≥ *Q*_max_
0	0–1	1	0–1	0
unsustainable	moderately sustainable	sustainable	moderately sustainable	unsustainable

a *Q*_min_ and *Q*_max_ represent the minimum and maximum streamflow rates under preimpact conditions. MMS denotes the median monthly streamflow. *Q*_25_ and *Q*_75_ indicate the lower and upper quartiles of the natural flow conditions, respectively. Although intermediate scores are grouped under the category “moderately sustainable”, the continuous indicator value preserves information on the magnitude of deviation from the natural regime; e.g., scores of 0.1 and 0.9 both fall within this category but represent substantially different sustainability states.

### 2.6 Building WEAP–LEAP Model for the 21st Century

The impacts of climate change and socioeconomic development on the WEFE Nexus were evaluated over 2020–2100 using three time slices: near century (2020–2030), midcentury (2055–2065), and far century (2090–2100). To minimize model spin-up effects, the first two years of each time slice were treated as a warm-up period and excluded from indicator calculations; therefore, the reported indicator values represent 2022–2030, 2057–2065, and 2092–2100, respectively.

Climate projections were derived from dynamically downscaled WRF simulations with ensemble means calculated separately for RCP 4.5 and RCP 8.5. These were incorporated as distinct climate scenarios at the subbasin level. Socioeconomic assumptions were aligned with SSP1, SSP2, and SSP5 to ensure consistency with the selected climate pathways. Figure S1 illustrates key features of the SSPs, including trends in population, economic development, education, urbanization, and technological change. Among these, SSP1 reflects the most sustainable trajectory, emphasizing environmental protection, resource efficiency, and the adoption of renewable energy.^48^ SSP3 was excluded due to its assumptions of widespread poverty and regional conflict,^65^ while SSP4 was omitted because its emphasis on global inequality was considered less relevant at the basin scale.^66^ SSP5 was retained as it is the only socioeconomic pathway consistent with RCP 8.5.^67^

Figure S2 illustrates the scenario hierarchy implemented in the WEAP–LEAP model. For this study, data modifications were based primarily on national-, regional-, or basin-scale sources. When such data were unavailable, projections from the SSP database^68^ at the R5.2OECD regional level, including Türkiye, were used. To ensure consistency, relative percent changes were applied proportionally to the Sakarya Basin’s baseline conditions. The following nine data categories were adjusted for each scenario:

1 Climate: daily time series of precipitation, temperature, wind speed, and relative humidity were modified based on downscaled ensemble projections.
2 Land use: land use distributions were adjusted using SSP-based regional projections^65,68^ and mass-balance corrections to preserve basin area. For example, irrigated area growth rates varied from 0.06%/year (SSP1) to 0.6%/year (SSP5), consistent with low- and high-growth assumptions.
3 Municipal water demand: future demand was estimated based on global sectoral trends. Under SSP2, municipal withdrawals were projected to increase by 56.5% in 2050 and 63.3% in 2100, relative to 2010 levels.^69^
4 Industrial water demand: similarly, industrial withdrawals were projected to increase by 52.4% by 2050 under SSP2, while SSP1 assumed a 45.2% decrease by 2100, reflecting water efficiency improvements.
5 Energy demand: basin-scale electricity generation was projected using current, under-construction, and planned power plants. In SSP1, full deployment of solar (3681 MW) and wind (1307 MW) capacity was assumed by 2100, reflecting a strong renewable energy focus.
6 Cooling systems in thermal power plants: water consumption factors varied by cooling technology. For example, lignite plants with wet cooling consumed 2600.6 m^3^/GWh, while dry systems required only 97.2 m^3^/GWh. SSP1 assumed a complete transition to dry cooling by 2100.
7 Municipal water losses: based on Turkish regulations, losses were assumed to decline to 25% by 2028 in SSP2 and SSP5. In SSP1, they were further reduced to 0% by 2100.
8 Treated wastewater reuse: reuse rates increased from 5% in 2023 to 15% in 2030 across all scenarios, but SSP1 extended this to 30% by 2100.
9 Environmental flow requirements: environmental flow allocations were only enforced in SSP1, using naturalized flow data at each subbasin outlet and assigned the highest priority in the WEAP allocation algorithm.

Detailed sources, assumptions, and quantitative inputs related to future land use, water demand, energy generation, and infrastructure are provided in the Supporting Information (Tables S3–S9; Figure S3). These comprehensive modifications allowed the WEAP–LEAP model to reflect spatially explicit, scenario-responsive system behavior across water, energy, food, and ecosystem domains throughout the 21st century.

### 2.7 Aggregation of WEFE Nexus Indicators

After calculating all 17 indicators across the seven subbasins using outputs from the WEAP–LEAP modeling framework, a multistep aggregation process was used to construct the WEFE Nexus Index. This process aims to capture not only best- and worst-case scenarios but also the system’s relative distance from defined sustainability thresholds.

First, the indicator values were normalized using continuous linear functions, enabling comparability across different units and scales.^70,71^ Each indicator’s score ranges from 0 (unsustainable) to 1 (fully sustainable), with sustainability thresholds informed by SDG targets, Türkiye’s energy and climate commitments, and natural hydrologic variability. For instance, 100% coverage was set as the optimal target for municipal water demand coverage, IDM, and hydropower production as % of maximum hydropower generation capacity, while CO_2_ emission reduction targets varied by period (50% near century, 100% mid/far century), and RES targeted 30% based on national policy. Ecosystem indicator scores were derived by comparing monthly median streamflows (MMS) to the IQR of naturalized flows (Table 2).

Once normalized, indicator scores within each pillar were aggregated using the arithmetic mean to obtain four distinct pillar scores: water, energy, food, and ecosystem. Equal weighting was applied at the pillar level, reflecting the integrated and multicentric philosophy of the WEFE Nexus approach. As a result, pillars containing a larger number of indicators (e.g., ecosystem) contributed equally to the index as other pillars, but each of their individual indicators carried a smaller weight in the final WEFE Nexus Index (Table 3).

**Table 3:** Weight Distribution of the WEFE Nexus Indicators and Pillars

indicator no	indicator code	indicator weight in the index	Pillar	pillar weight in the index
1	MDC	0.250	Water	0.25
2	IDM	0.250	Food	0.25
3	HPP_MGC	0.083	Energy	0.25
4	CO_2__EG	0.083		
5	RES	0.083		
6	Jan-MMS	0.021	Ecosystem	0.25
7	Feb-MMS	0.021		
8	Mar-MMS	0.021		
9	Apr-MMS	0.021		
10	May-MMS	0.021		
11	Jun-MMS	0.021		
12	Jul-MMS	0.021		
13	Aug-MMS	0.021		
14	Sep-MMS	0.021		
15	Oct-MMS	0.021		
16	Nov-MMS	0.021		
17	Dec-MMS	0.021		

Finally, the four pillar scores were averaged using the arithmetic mean to calculate the overall WEFE Nexus Index. This method was selected for its simplicity, transparency, and alignment with similar sustainability indices, such as the SDG Index and Human Development Index.

## 3 Results and Discussion

The WEFE Nexus assessment is grounded in a calibrated and validated WEAP–LEAP modeling framework applied across seven subbasins of the Sakarya Basin. Model performance statistics and calibration details are provided in Özcan (2023),^41^ with supplementary metrics and spatial references presented in the Supporting Information (Table S2 and Figure S4).

A baseline scenario representing observed climatic and socioeconomic conditions from 2004–2017 serves as the reference point for assessing future sustainability trajectories. This reference case captures the dynamics of existing infrastructure, land use, water demand, and energy production, allowing deviations under future scenarios to be interpreted as systemic shifts away from current resource interactions. In this way, the baseline anchors the analysis not only in past conditions but also in the evolving interdependencies that define the Sakarya’s coupled water–energy–food–ecosystem.

Figure 4 illustrates the performance of each WEFE Nexus pillar (water, energy, food, and ecosystem) across SSP1, SSP2, and SSP5, and Figure 5 presents the aggregated Nexus Index values. The exact numerical values of the WEFE Nexus Index are provided in Figure S5. These comparisons highlight not only numerical variation but also the systemic consequences of alternative development trajectories. Across most subbasins, SSP1 delivers the highest Nexus Index values, reflecting the benefits of sustainability-oriented socioeconomic pathways. Göksu is a notable exception: here, WEFE performance remains relatively stable across futures, suggesting that local resilience factors may buffer the subbasin against wider systemic pressures.

**Figure 4:**
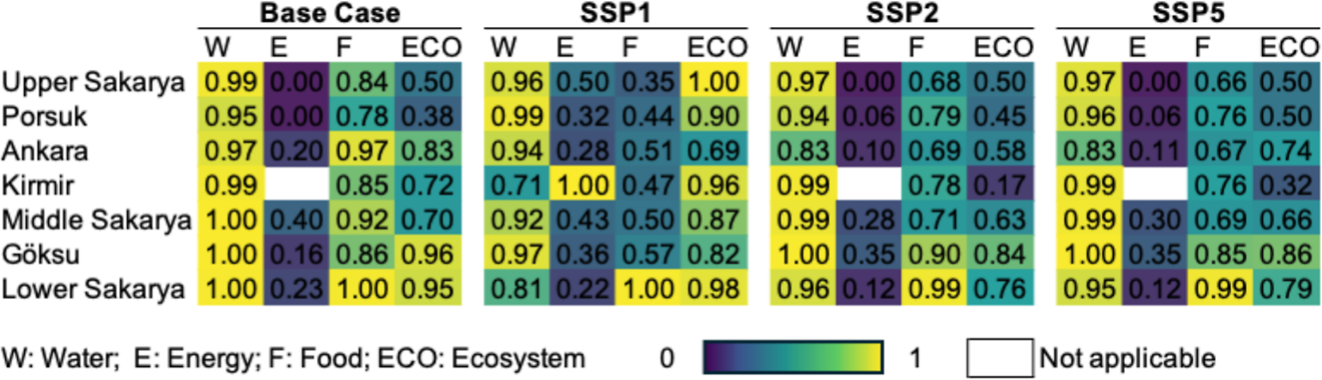
Pillar scores of all subbasins in the 21st century.

**Figure 5:**
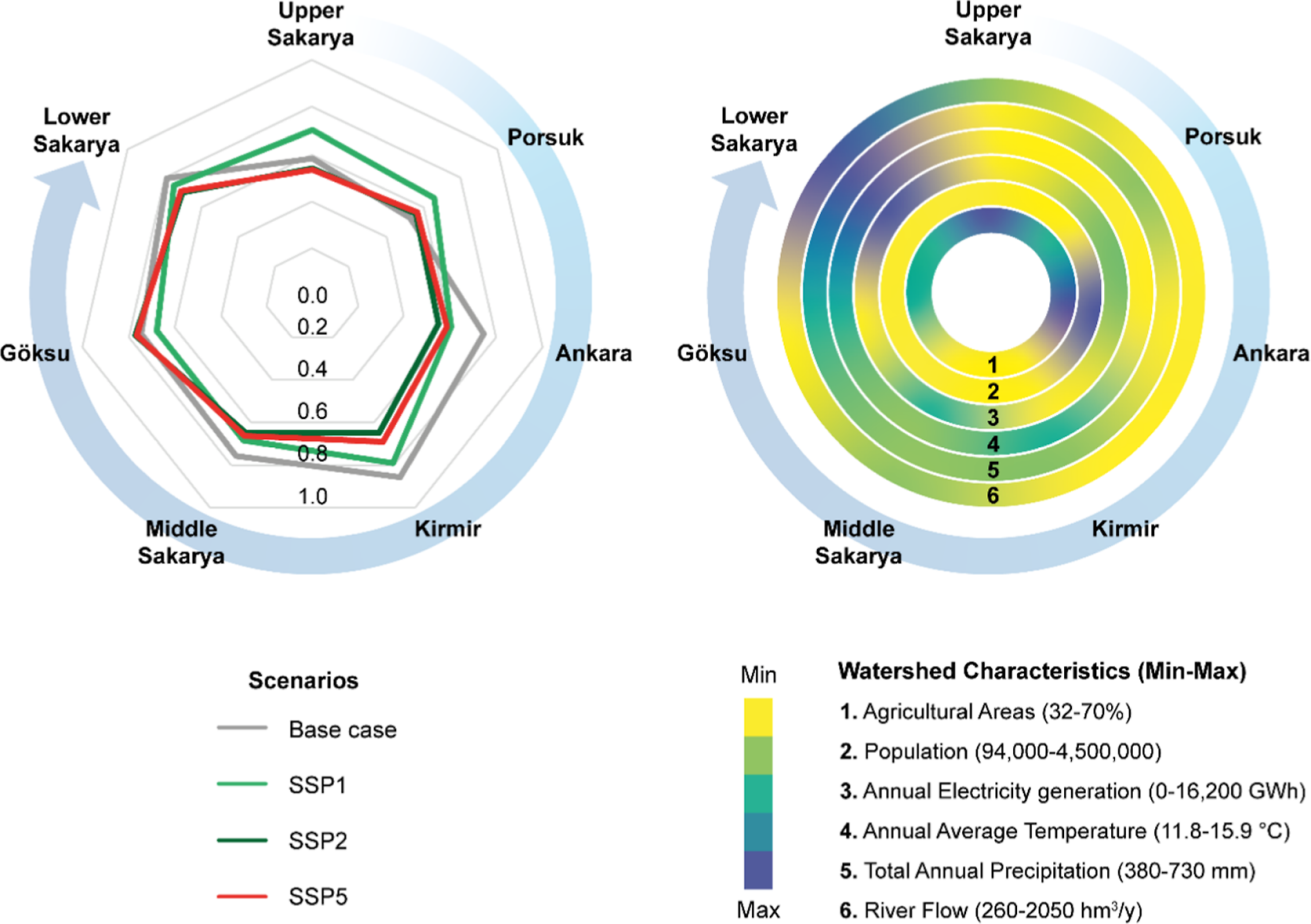
Comparison of WEFE Nexus Index across subbasins in the Sakarya Basin under different scenarios in the 21st century. The radar chart (left) compares WEFE Nexus Index values across subbasins under the Base Case and scenarios SSP1, SSP2, and SSP5. The concentric circle chart (right) depicts watershed characteristics, including agricultural areas, population, electricity generation, temperature, precipitation, and river flow, scaled from minimum to maximum values.

In contrast, SSP2 consistently generates the lowest scores, exposing the risks of continuing current practices that privilege short-term economic growth while neglecting environmental and social dimensions. Notably, the convergence of SSP2 and SSP5 outcomes across the basin suggests that incremental siloed approaches may yield systemic outcomes nearly as unsustainable as those under high-emission futures. This highlights the need for targeted, cross-sector interventions and transformative policies to move the system beyond these entrenched paths.

An important finding is that in several subbasins, including Ankara, Kirmir, Middle Sakarya, and Lower Sakarya, the Base Case outperforms even the sustainability-oriented SSP1. This apparent paradox underscores the dominant role of climate pressures in shaping future nexus performance. While SSP1 assumes progressive socioeconomic trajectories, it is also tied to more severe climate impacts than those experienced under the present-day baseline, illustrating how favorable social pathways alone cannot offset systemic vulnerability to climatic change.

Subbasin-level variation further highlights the influence of the local characteristics. Kirmir and Göksu consistently achieved the highest scores across scenarios. Both subbasins are characterized by relatively low population densities and moderate development pressures. Kirmir functions primarily as a drinking water basin with limited agricultural stress, while Göksu, despite its agricultural and industrial activities, benefits from less severe climate change impacts. Conversely, Upper Sakarya frequently records the lowest Nexus Index values. Despite its upstream location and limited external pressures, it is the driest subbasin, and projected climatic conditions coupled with agricultural demands pose persistent sustainability challenges. On the other hand, Lower Sakarya, the most downstream subbasin, achieves high nexus scores despite cumulative upstream impacts, largely due to more favorable climatic conditions.

To enhance interpretation, Figure 6 categorizes pillar scores by performance level, enabling complex, multiscenario results to be synthesized into clear decision signals. This visualization makes systemic imbalances across pillars and subbasins more visible: high scores (0.9–1.0) represent areas approaching sustainability, medium scores (0.5–0.8) highlight sectors at risk, and low scores (0.0–0.4) reveal acute vulnerabilities requiring urgent intervention.

**Figure 6:**
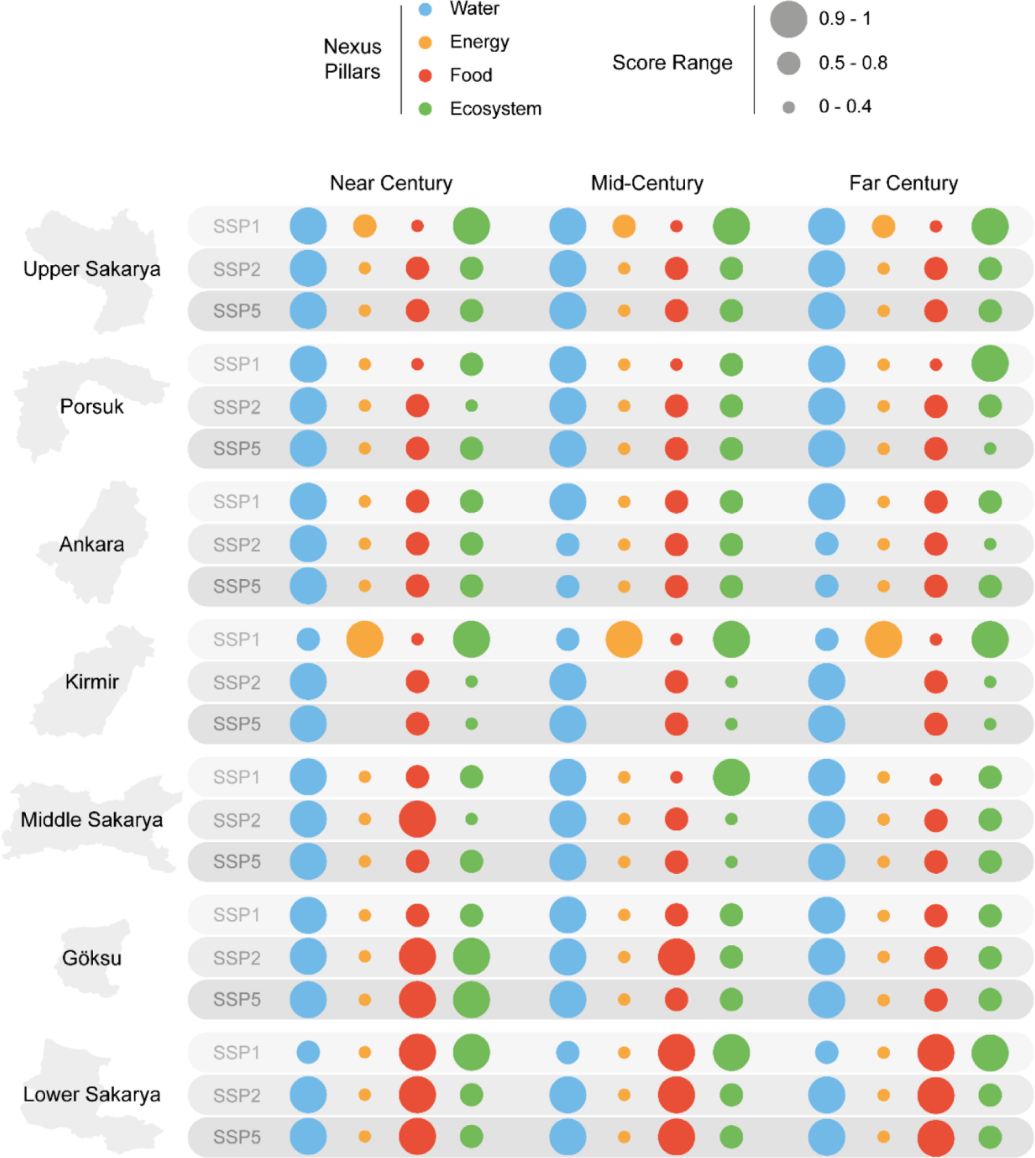
Comparative analysis of categorized pillar scores under different scenarios (SSP1, SSP2, and SSP5). Circle color represents the four nexus pillars (blue - water; orange - energy; red - food; and green - ecosystems), while circle size represents the WEFE Nexus score for each pillar.

The disaggregated results underscore how systemic pressures manifest differently across subbasins. In Upper Sakarya, persistently low agricultural scores are driven by scarce precipitation and high irrigation demand, illustrating how climate constraints create chronic trade-offs between food security and ecosystem flows. Porsuk faces similar agricultural challenges but is further undermined by declining ecosystem scores, reflecting cumulative stress from agriculture and industry. In Ankara, high population and industrial activity generate both municipal water stress and agricultural deficits, demonstrating the limits of infrastructure-focused solutions in the absence of cross-sector adaptation. Kirmir, as a drinking water source with low cropland density, experiences fewer agricultural pressures but faces ecosystem flow challenges that threaten long-term resilience. Middle Sakarya’s relatively balanced land use is destabilized by climate-driven precipitation decline, showing how climatic shifts can destabilize otherwise balanced systems. Göksu, though buffered by stable precipitation, is increasingly stressed by rising temperatures, highlighting the value of the efficiency gains in irrigation. Lower Sakarya consistently achieves high Nexus scores despite cumulative upstream pressures, supported by favorable rainfall and lower climate vulnerability. Yet this apparent resilience is fragile: the subbasin’s reliance on thermal power compromises energy sustainability and links its long-term trajectory to broader decarbonization policies. These patterns reinforce the need for subbasin-specific strategies that recognize localized vulnerabilities while ensuring basin-wide coherence. The similarity of SSP2 and SSP5 outcomes in several subbasins further illustrates that business-as-usual development may deliver sustainability outcomes nearly as poor as those under fossil-intensive futures. By contrast, SSP1, while not universally optimal, demonstrates the greatest systemic balance, improving outcomes across all four pillars, with average scores of approximately 0.9 for water, 0.44 for energy, 0.55 for food, and 0.89 for ecosystem across subbasins.

Temporal patterns across the three future windows reveal both stability and divergence in the nexus pillar performance. Notably, water scores remain relatively stable over time in most subbasins, suggesting that the infrastructure and allocation mechanisms modeled within the WEAP may buffer the water sector against intensifying climate stress. In contrast, the ecosystem and food pillars show significant declines from the near future to the far future, especially under SSP2 and SSP5, highlighting increased stress from competing demands, flow alterations, and rising temperatures. For example, in Kirmir, water scores remain high across scenarios and times, even as ecosystem and food scores decline. This suggests that ensuring water availability alone does not guarantee ecological or agricultural sustainability without explicit environmental flow and irrigation efficiency measures. Similarly, Middle Sakarya maintains a strong water performance despite projected precipitation declines, which may be attributed to reservoir storage or seasonal shifts that partially offset reduced precipitation. In contrast, SSP1 demonstrates a more balanced and adaptive evolution over time, with several subbasins maintaining or improving scores across pillars.

These findings directly inform the “Response” component of the PSR-based WEFE Nexus framework introduced in Figure 2. For example, consistently low scores in the food and ecosystem pillars in Upper Sakarya and Porsuk highlight the need for infrastructure development and upgrades, such as enhancing irrigation efficiency and securing environmental flows. The energy challenges in Lower Sakarya, where dependence on thermal power hinders sustainability, indicate a need for financial incentives and support to encourage renewable energy adoption and diversify the grid. In subbasins like Ankara, where cross-sector trade-offs are most pronounced, integrated policies are critical to managing competing water, energy, and food demands. Subbasins with moderate scores across all pillars may benefit from public awareness and education campaigns to foster behavioral change and demand-side management. Lastly, the systemic patterns identified by the Nexus Index underscore the importance of research and innovation for adaptive, region-specific solutions and highlight the value of international collaboration to share best practices in similar hydroclimatic contexts.

Overall, the findings show that while municipal water supply targets are largely achievable, ecosystem integrity and renewable energy goals remain persistently out of reach without deliberate, cross-sector intervention. Crucially, no scenario produces a consistently high performance across all four pillars. This reflects the systemic trade-offs and spatial heterogeneity inherent in the WEFE Nexus: improvements in one domain often come at the expense of another, and localized resilience may coexist with vulnerabilities elsewhere. Even sustainability-oriented trajectories such as SSP1 cannot overcome these dynamics in the absence of integrated management. These results highlight a central insight: resilience cannot be achieved through siloed or incremental measures but requires spatially differentiated, climate-aware, and cross-sector policy frameworks that address systemic interdependencies.

Beyond the Sakarya Basin, the patterns identified in this study reflect structural dynamics relevant to many semiarid and Mediterranean-type river basins facing water stress, agricultural intensification, and energy transition under climate change. The convergence of SSP2 and SSP5 outcomes, for example, highlights the broader risk of “locked-in” unsustainability when incremental, sector-based planning prevails over integrated governance, a challenge also noted in other nexus contexts. Similarly, the trade-offs found among agricultural water use, ecosystem integrity, and energy production reveal systemic tensions common in regulated basins where hydropower, irrigation, and environmental flows compete for limited resources. Importantly, though the numerical results are basin-specific, the analytical approach combining coupled WEAP–LEAP modeling, scenario-based temporal windows, normalized multipillar indicators, and subbasin-scale diagnostics offers a transferable framework for assessing WEFE interconnections in regions with similar hydroclimatic and socioeconomic challenges. Thus, this study not only provides local insights for Sakarya but also establishes a decision-support approach for integrated resource planning in water-stressed basins worldwide.

Long-horizon, fully integrated WEFE Nexus assessments remain rare, but several recent studies provide useful points of comparison. Trade-offs between hydropower expansion and environmental objectives in the Himalayas have been documented,^66^ a tension that echoes in our findings, where energy development compromises ecological integrity. Assessments of the Brahmaputra Basin showed that stronger environmental safeguards can foster stakeholder cooperation,^72^ aligning with our conclusion that locally adapted, context-sensitive strategies enhance systemic resilience. Irrigation efficiency and environmental safeguards have similarly been emphasized as critical for agricultural resilience in Canada,^73^ consistent with our results in water-scarce subbasins. Integrated adaptation and cross-sector interventions in the Mediterranean further reinforce the value of a holistic nexus perspective.^74^

Building on this body of work, our framework contributes several novel elements that extend the nexus approach from conceptual framing to operational application. First, we incorporate three distinct future time horizons to capture near-, mid-, and long-term sustainability dynamics. Second, the framework is spatially disaggregated, enabling systemic vulnerabilities and resilience hotspots to be revealed at the subbasin level rather than masked by basin averages. Third, ecosystems are embedded as a fully quantified, coequal pillar through hydrological regime alteration metrics, moving beyond proxy-based or descriptive approaches. These elements are embedded within a coupled WEAP–LEAP platform, calibrated to the Sakarya Basin, and supported by dynamically downscaled climate projections and SSP-based socioeconomic pathways.

While results are consistent with recognized patterns, such as trade-offs between energy and environmental objectives, they also reveal spatial and temporal variation in resilience that is not evident in basin-aggregated studies. At the same time, limitations remain. Ensemble-averaged climate projections cannot capture the full uncertainty of future trajectories; future work should consider probabilistic scenarios, richer ecosystem representation (e.g., water quality and river morphology), and dynamic surface–groundwater interactions. Methodologically, the framework can be adaptable to updated climate projections (e.g., CMIP6) and can be enhanced through stakeholder participation in indicator threshold-setting and weighting. Taken together, these advances support the evolution of nexus assessments into transparent, transferable, and decision-oriented tools that embody systems thinking in practice.

Overall, the analysis shows that sustainability cannot be secured through isolated sectoral measures but requires integrated strategies that account for cross-sector feedback, ecosystem needs, and long-term climate and socioeconomic change. By operationalizing the WEFE Nexus into a transparent and transferable framework, this study provides actionable insights into designing adaptive policies that reconcile competing demands, safeguard ecosystems, and strengthen resilience across interconnected environmental systems.

## Supplementary Material



## Data Availability

All downscaled and bias-corrected temperature and precipitation projection data sets used in this study are openly available on Zenodo at: 10.5281/zenodo.17878703

## Notes

WEAP model calibration parameters across land-use classes; calibration and validation statistics for streamflow gauging stations in each subbasin; scenario-based estimates for future land use, irrigated area growth, and municipal and industrial water withdrawals; list and characteristics of existing, planned, and under-construction power plants in the Sakarya Basin; water consumption factors for thermal power plants; projected municipal water loss rates for SSP scenarios; key SSP characteristics adopted from O’Neill et al. (2017); hierarchy of climate and socioeconomic scenarios used in the WEAP–LEAP model; future electricity generation projections for the basin; WEAP catchments and locations of weather and streamflow stations; and pillar-level scores and WEFE Nexus Index trajectories for all subbasins throughout the 21st century. All calculated WEFE nexus indicator score data sets generated in this study are openly available on Zenodo at: 10.5281/zenodo.17879445 (PDF)
