# Supplementary material for: Uncovering Systemic Dynamics through an Integrated WEFE Nexus Index across 21st Century Futures
Source: Environ Sci Technol. 2026 Feb 5;60(6):4776–89. doi: 10.1021/acs.est.5c11740 (PMC12930377; doi:10.1021/acs.est.5c11740)
Supplement: Supplementary file 1 [file es5c11740_si_001.pdf]

Supplementary Information (SI) for

Uncovering Systemic Dynamics through an Integrated WEFE Nexus Index across 21st Century Futures

Zeynep Özcan[1], Emre Alp[1]*

[1]Department of Environmental Engineering, Middle East Technical University, Ankara, Türkiye,
[*]e-mail: zozcan@metu.edu.tr

Supplemental Notes. Tables S1-S9. Figures S1-S5. 8 pages

Table S.1. The list of calibration parameters used in the WEAP model calibration

Name of the Parameter	Land Use Class	Default Value	Unit	Calibration Range
Soil Water Capacity	Artificial surfaces	1000	mm	0 - 10000
	Non-irrigated arable land	1000	mm	0 - 10000
	Permanently irrigated land	1000	mm	0 - 10000
	Vineyards	1000	mm	0 - 10000
	Fruit trees and berry plantations	1000	mm	0 - 10000
	Pastures	1000	mm	0 - 10000
	Complex cultivation patterns	1000	mm	0 - 10000
	Land princ. occup. by agr., with signf. areas of natural veg.	1000	mm	0 - 10000
	Forests	1000	mm	0 - 10000
	Natural grasslands	1000	mm	0 - 10000
	Sclerophyllous vegetation	1000	mm	0 - 10000
	Transitional woodland-shrub	1000	mm	0 - 10000
	Bare rocks	1000	mm	0 - 10000
	Sparsely vegetated areas	1000	mm	0 - 10000
	Wetlands	1000	mm	0 - 10000
	Water bodies	1000	mm	0 - 10000
Runoff Resistance Factor (RRF)	Artificial surfaces	8.00	-	0 - 100
	Non-irrigated arable land	4.22	-	0 - 100
	Permanently irrigated land	4.22	-	0 - 100
	Vineyards	4.22	-	0 - 100
	Fruit trees and berry plantations	4.22	-	0 - 100
	Pastures	2.50	-	0 - 100
	Complex cultivation patterns	4.22	-	0 - 100
	Land princ. occup. by agr., with signf. areas of natural veg.	4.22	-	0 - 100
	Forests	5.18	-	0 - 100
	Natural grasslands	2.50	-	0 - 100
	Sclerophyllous vegetation	2.08	-	0 - 100
	Transitional woodland-shrub	2.08	-	0 - 100
	Bare rocks	1.31	-	0 - 100
	Sparsely vegetated areas	1.31	-	0 - 100
	Wetlands	6.34	-	0 - 100
	Water bodies	0.1	-	0 - 100
Preferred Flow Direction	Artificial surfaces	0.15	-	0 - 1
	Non-irrigated arable land	0.15	-	0 - 1
	Permanently irrigated land	0.15	-	0 - 1
	Vineyards	0.15	-	0 - 1
	Fruit trees and berry plantations	0.15	-	0 - 1
	Pastures	0.15	-	0 - 1
	Complex cultivation patterns	0.15	-	0 - 1
	Land princ. occup. by agr., with signf. areas of natural veg.	0.15	-	0 - 1
	Forests	0.15	-	0 - 1
	Natural grasslands	0.15	-	0 - 1
	Sclerophyllous vegetation	0.15	-	0 - 1
	Transitional woodland-shrub	0.15	-	0 - 1
	Bare rocks	0.15	-	0 - 1
	Sparsely vegetated areas	0.15	-	0 - 1
	Wetlands	0.15	-	0 - 1
	Water bodies	0.15	-	0 - 1
Root Zone Conductivity	Artificial surfaces	20	mm/month	0 - 1000
	Non-irrigated arable land	20	mm/month	0 - 1000
	Permanently irrigated land	20	mm/month	0 - 1000
	Vineyards	20	mm/month	0 - 1000
	Fruit trees and berry plantations	20	mm/month	0 - 1000
	Pastures	20	mm/month	0 - 1000
	Complex cultivation patterns	20	mm/month	0 - 1000
	Land princ. occup. by agr., with signf. areas of natural veg.	20	mm/month	0 - 1000
	Forests	20	mm/month	0 - 1000
	Natural grasslands	20	mm/month	0 - 1000
	Sclerophyllous vegetation	20	mm/month	0 - 1000
	Transitional woodland-shrub	20	mm/month	0 - 1000
	Bare rocks	20	mm/month	0 - 1000
	Sparsely vegetated areas	20	mm/month	0 - 1000
	Wetlands	20	mm/month	0 - 1000
	Water bodies	20	mm/month	0 - 1000
Deep Water Capacity	-	10000	mm	0 - 300000
Deep Conductivity	-	20	mm/month	0.1 - 150

Name of the Parameter	Land Use Class	Default Value	Unit	Calibration Range
Initial_Z2	-	30	%	0 - 100
Freezing Point	-	-5	℃	-20 - +20
Melting Point	-	+5	℃	-20 - +20

Table S.2. Summary statistics for streamflow gauging stations during model calibration and validation in each subbasin. Please see Özcan (2023) for details.

		Calibration			Validation		
**Subbasin Name**	**Station ID**	**NSE**	**PBIAS**	**R^2**	**NSE**	**PBIAS**	**R^2**
Upper Sakarya	E12A052	0.57	17.4	0.73	0.62	-1.7	0.64
Porsuk	E12A003	0.36	-31.6	0.64	0.60	-48.3	0.90
Ankara	D12A242	0.57	14.5	0.58	0.49	-7.1	0.50
Kirmir	D12A243	0.46	5.3	0.47	0.07	41.7	0.35
Middle Sakarya	E12A058	0.62	12.5	0.76	0.35	15.6	0.43
Göksu	E12A022	0.43	28.8	0.52	0.35	15.6	0.45
Lower Sakarya	E12A057	0.61	15.7	0.72	0.55	19.2	0.68

Table S.3. Sources for the future land use area estimates

Land Use Class	Reference
Forest	SSP web-database (IIASA, 2018)
Built-up Area	SSP web-database (IIASA, 2018)
Pasture	SSP web-database (IIASA, 2018)
Cropland	SSP web-database (IIASA, 2018)
Irrigated Areas	Hanasaki et al. (2013)

Table S.4. Irrigated area growth change per scenario (Hanasaki et al., 2013)

Scenario	Irrigated area growth ($\%y^{-1}$)
SSP1	0.06
SSP2	0.3
SSP5	0.6

Table S.5. Percent change in the global municipal water withdrawals as compared to the year 2010

2050			2100		
SSP1	***SSP2***	***SSP5***	***SSP1***	***SSP2***	***SSP5***
55.1	56.5	72.2	27.4	63.3	70.5

Table S.6. Percent change in the global industrial water withdrawals as compared to the year 2010 (Graham et al., 2018).

**2050**			**2100**		
SSP1	***SSP2***	***SSP5***	***SSP1***	***SSP2***	***SSP5***
6.0	52.4	38.9	-45.2	39.5	-9.9

Table S.7. List of power plants under construction or planned to be built in the Sakarya Basin

**Name of the power plant**	**Type**	**Province**	**District**	**Subbasin**	**Installed Power (MW)**	**Electricity Generation (GWh)**	**Actual Realization (%)**
Gürsöğüt HPP	Hydroelectric	Eskişehir	Mihalıççık	Middle Sakarya	242.0	158.7	91
Kargı HPP	Hydroelectric	Ankara	Beypazarı	Middle Sakarya	100.0	203.0	100
Bozüyük WPP	Wind	Bilecik	Bozüyük	Middle Sakarya	90.0	323.0	90
Adapazarı WPP	Wind	Bolu	Göynük	Middle Sakarya	80.0	70.0	96
WPP YEKA-1	Wind	Eskişehir	Tepebaşı	Porsuk	50.0	175.0	No info
Kartal WPP	Wind	Eskişehir	Tepebaşı	Porsuk	39.0	136.5	93
Arıkçayırı BPP	Biomass	Bolu	Göynük	Middle Sakarya	30.0	208.2	96
Meryem WPP	Wind	Bilecik	Merkez	Middle Sakarya	30.0	73.0	20
Kırka FOPP	Fuel-oil	Eskişehir	Kırka	Upper Sakarya	26.9	177.0	100
Pamukova WPP	Wind	Sakarya	Geyve	Middle Sakarya	20.0	70.0	98
Kuyulukoyak WPP	Wind	Konya	Sarayönü	Upper Sakarya	16.0	57.0	96
İnegöl BPP	Biomass	Bursa	İnegöl	Göksu	14.1	60.5	57
Hisar HPP	Hydroelectric	Bilecik	İnhisar	Middle Sakarya	13.4	11.9	0
Ova HPP	Hydroelectric	Sakarya	Pamukova	Middle Sakarya	13.2	30.0	72
Gök HPP	Hydroelectric	Bilecik	Osmaneli	Middle Sakarya	12.6	20.0	100
İnönü NGPP	Natural Gas	Eskişehir	İnönü	Porsuk	12.6	73.7	100
Boğazköy HPP	Hydroelectric	Eskişehir	Mihalıççık	Göksu	10.0	20.0	100

NGPP:Natural Gas Power Plant; HPP: Hydroelectric Power Plant; BPP: Biogas Power Plant; WPP: Wind Power Plant; FOPP: Fuel Oil Power Plant

Table S.8. Water consumption factors of the thermal power plants modeled in the study

**Fuel Type**	**Cooling Type**	**Water Consumption Factor (m^3/GWh)**	**Reference**
Lignite/Coal	Wet	2,600.6	(Macknick et al., 2012)
Lignite/Coal	Dry	97.2	(Spang et al., 2014)
Natural Gas	Wet	776.0	(Macknick et al., 2012)
Natural Gas	Dry	7.6	(Macknick et al., 2012)
Biogas	Wet	776.0	(Macknick et al., 2012)

Table S.9. Water losses in municipal water networks in the future scenarios

	2023	2028	2100
SSP1	30%	25%	0%
SSP2	30%	25%	25%
SSP5	30%	25%	25%

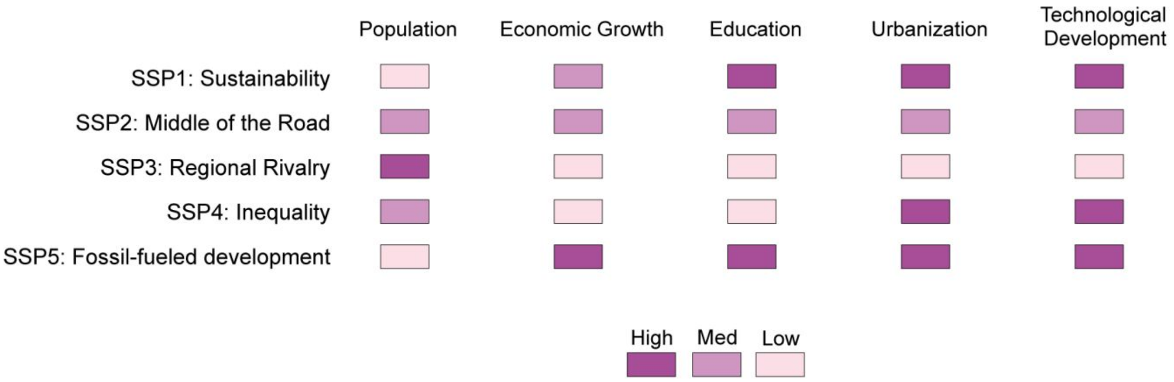

Figure S.1. Comparison of key features of SSPs with respect to population, economic growth, education, urbanization, and technological development. Adopted from O'Neill et al. (2017),[47] *SSP*: Shared Socioeconomic Pathway

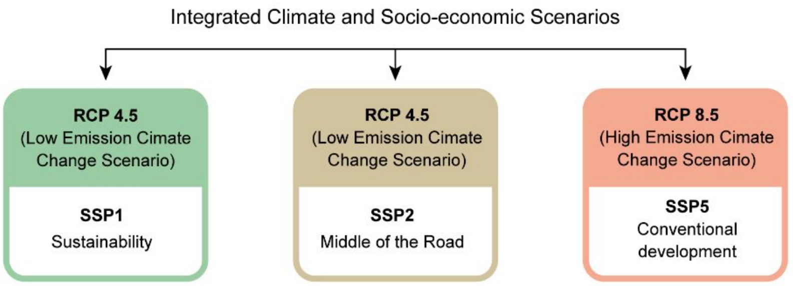

Figure S.2. Hierarchy of climate change and socioeconomic scenarios in the WEAP-LEAP model.

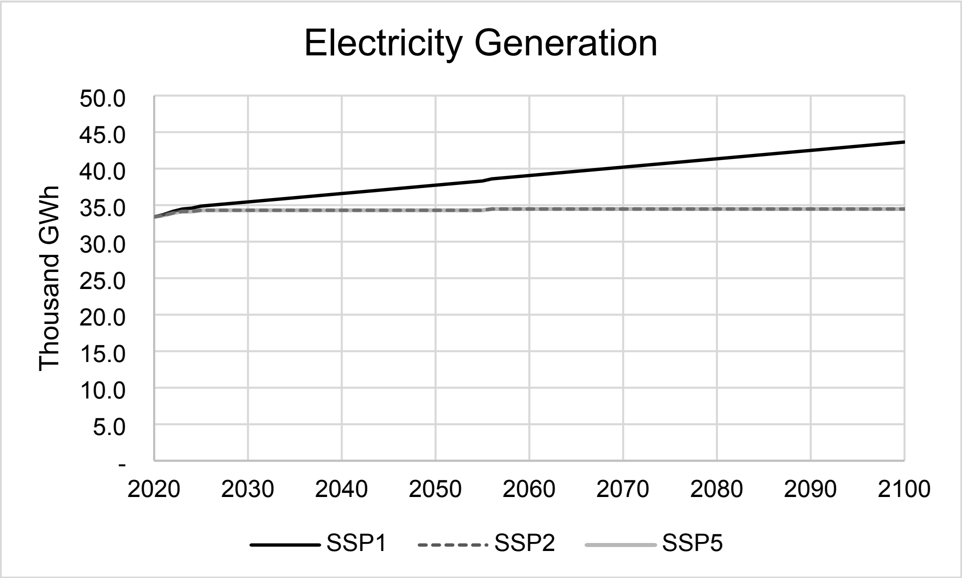

Figure S.3. Total electricity generation in Sakarya Basin for the future scenarios

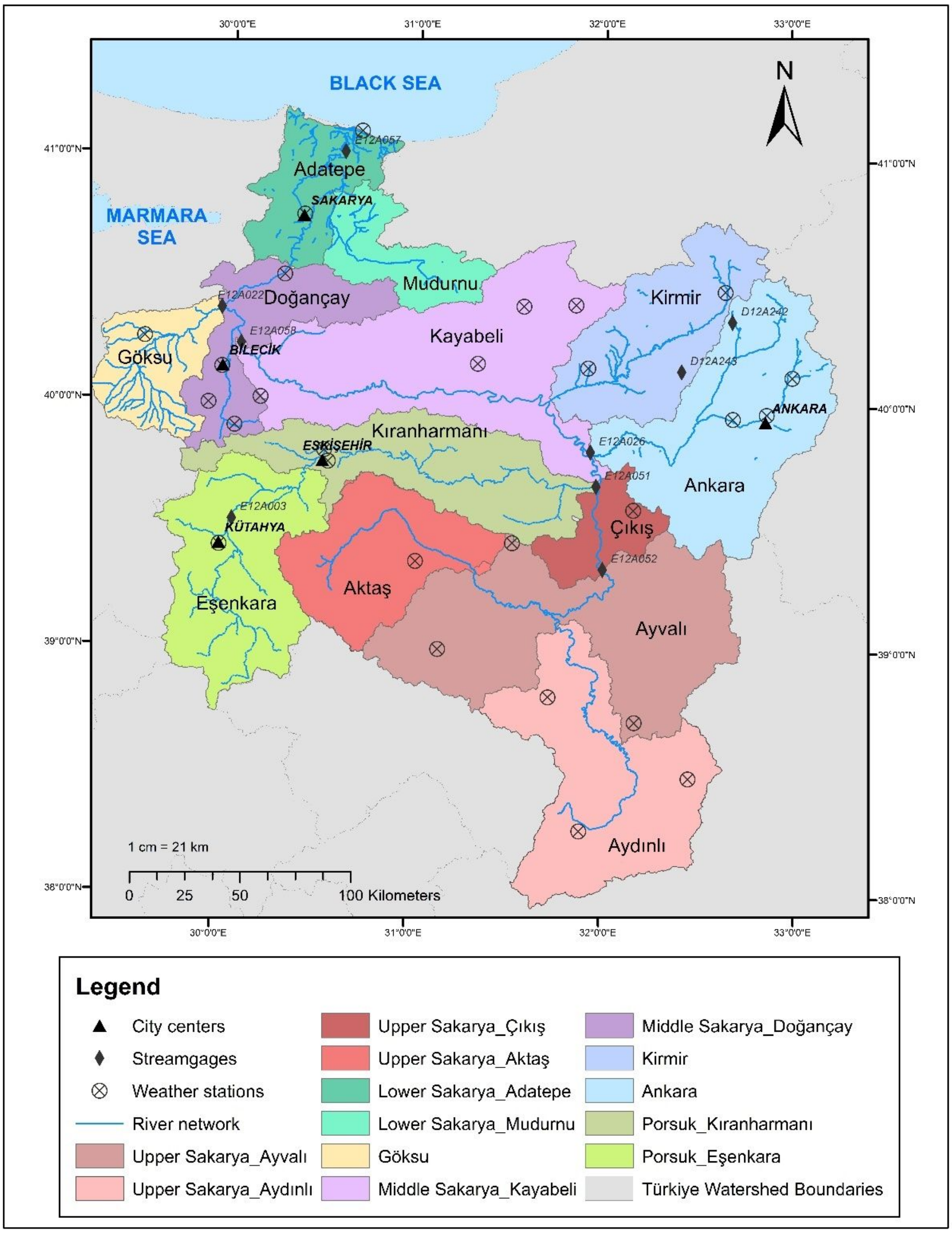

Figure S.4. WEAP catchments, locations of weather stations used for the model setup, and the locations of streamgages used in the model calibration

	Base Case					SSP1					SSP2					SSP5				
	W	E	F	ECO	*WEFE Nexus Index*	W	E	F	ECO	*WEFE Nexus Index*	W	E	F	ECO	*WEFE Nexus Index*	W	E	F	ECO	*WEFE Nexus Index*
Upper Sakarya	0.99	0.00	0.84	0.50	*0.58*	0.96	0.50	0.35	1.00	*0.70*	0.97	0.00	0.68	0.50	*0.54*	0.97	0.00	0.66	0.50	*0.53*
Porsuk	0.95	0.00	0.78	0.38	*0.53*	0.99	0.32	0.44	0.90	*0.66*	0.94	0.06	0.79	0.45	*0.56*	0.96	0.06	0.76	0.50	*0.57*
Ankara	0.97	0.20	0.97	0.83	*0.74*	0.94	0.28	0.51	0.69	*0.60*	0.83	0.10	0.69	0.58	*0.55*	0.83	0.11	0.67	0.74	*0.59*
Kirmir	0.99	-	0.85	0.72	*0.86*	0.71	1.00	0.47	0.96	*0.79*	0.99	-	0.78	0.17	*0.65*	0.99	-	0.76	0.32	*0.69*
Middle Sakarya	1.00	0.40	0.92	0.70	*0.75*	0.92	0.43	0.50	0.87	*0.68*	0.99	0.28	0.71	0.63	*0.65*	0.99	0.30	0.69	0.66	*0.66*
Göksu	1.00	0.16	0.86	0.96	*0.74*	0.97	0.36	0.57	0.82	*0.68*	1.00	0.35	0.90	0.84	*0.77*	1.00	0.35	0.85	0.86	*0.77*
Lower Sakarya	1.00	0.23	1.00	0.95	*0.79*	0.81	0.22	1.00	0.98	*0.75*	0.96	0.12	0.99	0.76	*0.71*	0.95	0.12	0.99	0.79	*0.71*

Figure S.5. Pillar scores and WEFE Nexus Index values of all subbasins in the 21st century
